# Image transmission and video live-streaming in emergency medical communication centre: a narrative review of its feasability, use, and impact

**DOI:** 10.1186/s13049-026-01552-1

**Published:** 2026-01-17

**Authors:** Nicolas Marjanovic, Sylvain Michaud-Monvoisin, Marie Dubocage, Matthieu Lassalle, Jérémy Guenezan, Olivier Mimoz

**Affiliations:** 1 CHU de Poitiers, Emergency Department and Prehospital Care, Rue de La Milétrie, Poitiers, 86000 France; 2 CHU de Poitiers, INSERM CIC1402 – IS-ALIVE, Rue de La Milétrie, Poitiers, 86000 France; 3 Université de Poitiers, Faculté de Médecine, Rue de La Milétrie, Poitiers, 86000 France; 4 Université de Poitiers, INSERM, Poitiers, U1070 PHAR2 France

**Keywords:** Telemedicine, Emergency Medical Services, Triage, Videoconferencing, Remote consultation

## Abstract

**Introduction:**

Emergency Medical Communication Centres (EMCC) are responsible for assessing calls, providing pre-arrival instructions, and dispatching resources. Telephone-based triage is limited by the lack of visual information, which may lead to under-triage or over-triage. Telemedicine, including image transmission and video live-streaming has emerged as a complementary tool, but its actual impact in EMCC remains uncertain. We aimed to summarise the existing literature on the feasibility, use and impact of telemedicine using image transmission or video live-streaming in EMCCs.

**Methods:**

We conducted a narrative review of the literature from January 2000 to July 2025 using Medline and Web of Science. Eligible studies were randomised controlled trials, prospective or retrospective observational, or qualitative studies conducted in EMCC or equivalent settings, assessing telemedicine through image transmission and video live-streaming. Simulation studies, case reports, reviews, and conference abstracts were excluded.

**Results:**

Twenty studies were included. Feasibility and acceptability were consistently high, with acceptance rates between 86% and 100%. Image transmission and video-live streaming improved situational awareness and provided reassurance for callers, though some dispatchers reported discomfort. Clinical applications included unspecified calls, altered consciousness, seizures, road traffic accidents, minor trauma, dyspnoea, nursing home resident, or when children are involved. Image transmission and video live-streaming were associated with reduced Emergency Medical Service and ambulance dispatching, improved patients orientation in mild trauma, fewer unnecessary transfers from nursing homes and better recognition of severity signs in dyspnoea. Impact on triage quality has yet to be formally demonstrated. Failure rate was reported to range from 2.4% and 30%, mainly due to poor network coverage or technical difficulties.

**Conclusion:**

Image transmission and video live-streaming for emergency triage appear feasible and promising in EMCC. It is generally well accepted by medical dispatchers, paramedics, and patients, who consider it useful. This approach may improve decisions made by medical dispatchers. However, its impact on the quality of triage still needs to be more formally assessed.

## Introduction

Emergency Medical Communication Centres (EMCC) are centralized facilities responsible for receiving emergency calls, playing a crucial role in emergency care. They allow assessment of the urgency and nature of medical situations, provide pre-arrival instructions, and coordinate the dispatch of appropriate healthcare resources, such as ambulances or mobile intensive care units. Although organizational models vary across countries, all EMCCs share common features: they must manage an increasing volume of calls, identify and locate the patient, and perform a quick clinical assessment by phone with the aim of providing an appropriate response [1–6].

Despite the essential role of EMCCs in emergency care, telephone-based triage faces inherent limitations due to the lack of visual information. Call-takers must base their assessment solely on what the caller describes, which is often fragmented, emotionally influenced, or lacking clinical accuracy [7]. These constraints create uncertainty in judging the seriousness of the situation and understanding the patient’s condition. As a result, the risk of incorrect interpretation is significant, exposing the triage process to both under-triage and over-triage. Under-triage—when a severe condition is not recognized—can lead to delayed treatment with direct consequences for the patient’s outcome, particularly in time-critical emergencies. Over-triage, by contrast, may result in the unnecessary mobilisation of advanced medical resources, leading to system overload, inefficient care distribution, reduced availability for patients in genuine need, and additional costs for both the patients and the community. These limitations reveal the structural vulnerability of telephone-based triage and the need for tools that enhance clinical judgement and resource allocation in remote settings.

Telemedicine is defined as all medical interventions performed remotely using information and communication technologies [8, 9]. It enables, in particular, the transmission of medical documents, electrocardiograms, and the conduct of remote consultations. Its implementation within EMCCs is of growing interest. In this setting, the use of image transmission or video live-streaming based triage may serve as complementary tools to telephone-based triage. However, the available evidence is mostly derived from observational, retrospective, or qualitative studies. To date, few randomised controlled trials have been conducted, and the actual impact of this method in the context of EMCC remains poorly understood [10–12].

In this narrative review, we aim to summarise the existing literature on the feasibility, use and impact of telemedicine using image transmission or video live-streaming in EMCCs. We discuss its potential benefits, identified limitations, and the practical and ethical challenges associated with its implementation.

## Methods

### Design

We conducted a narrative review with the aim of summarizing the existing literature on the use of telemedicine in EMCCs, specifically image transmission, referring to the remote sharing of still images captured via smartphone or dedicated devices, transmitted in real time or asynchronously, and video live-streaming, referring to the real-time transmission of moving images from the caller’s or responder’s device. Our approach is to provide an overview of the current state of knowledge, based on published studies.

### Study selection and inclusion criteria

We included randomised clinical trials, as well as prospective and retrospective observational studies and qualitative studies, if they met all of the following inclusion criteria: (1) conducted in an Emergency Medical Communication Centre (EMCC) or equivalent; (2) involved in the management of emergency calls; (3) assessed telemedicine using image transmission and video live-streaming; (4) including adult or paediatric patients and (5) published in English. We excluded simulation studies, case reports, conference abstracts, reviews and editorials. In addition, questionnaire-based studies were also excluded, as their declarative nature limits the ability to assess real-life use and impact of image transmission and video live-streaming.

### Search strategy

We conducted a comprehensive literature search of Medline and Web of Science database, using Medical Subject Heading and Free text terms, from January 2000 to July 2025. Reviewers also screened references of relevant articles to determine their suitability.

The search was performed using a combination of keywords and Boolean operators to identify relevant studies. The search strategy included MeSH terms and synonyms such as *“Telemedicine,” “Telehealth,” “eHealth,” “Mobile Health,” “Tele-Referral,” “Videoconferencing,” “Video Conference,” “Video Telecommunication,”* as well as truncated terms like *video* and *streaming*, in combination with *“Emergency Medical Service*,” “Emergency Dispatch*,” “Emergency Medical Call*” and “Emergency Medical Communication*.”

Identified studies were screened by title and abstract to assess their eligibility. For each eligible study, two reviewers summarised the following: (1) study characteristics; (2) year of publication; (3) sample size; (4) participant’s characteristics; (5) type of software used; (6) main objective and (7) main findings. Conflicts required the involvement of a third reviewer for the manuscript analysis. The results were then categorized based on themes such as feasibility, usability, clinical applications, limitations and ethical considerations. We performed a qualitative synthesis of the findings to summarize the key themes emerging from the literature.

## Results

**Fig. 1:**
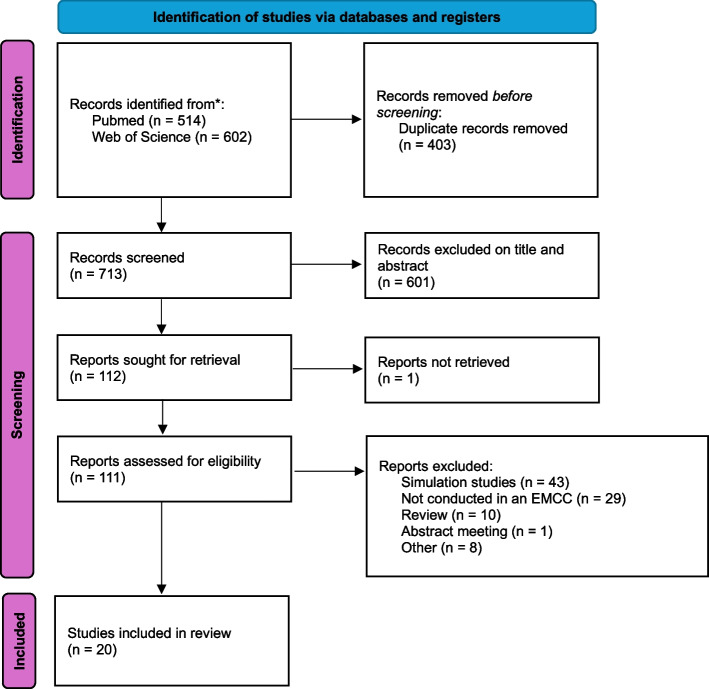
Flow-diagram of search procedure

A total of 1,116 potentially relevant publications were identified through database analysis (Fig. 1). Of these, 403 (36.2%) were excluded as duplicates, 601 (53.9%) based on title and abstract screening, 43 (3.9%) for being simulation studies, 29 (2.6%) for not being conducted within an Emergency Medical Communication Centre (EMCC) or equivalent setting, 10 (1%) reviews, and one (0.1%) abstract meeting. In addition, one reports has not been retrieved, and 8 (0.7%) were excluded for other reasons, and. Ultimately, 20 studies (1.6%) were included in the analysis. Their characteristics are presented in Table 1.

**Table 1:** Characteristics and results of qualitative and clinical studies assessing telemedicine using video or photographic transmission in an EMCC

**Author**	**Year**	**Design**	**Primary objective**	**Primary outcome**	**Software**	**On-site contact**	**Population**	**Size**	**Main results**
Bergrath	2013	Pilot study	to analyse the feasibility and possible limitations of prehospital teleconsultation during the implementation phase of this system.	not specified	not specified	Ambulance	not specified	296 calls35 video-calls	No patients refused video-callsDuration of calls : 24,9 min
Bergrath	2013	Pilot study	to investigate the feasibility of using a digital camera for still picture transmission in a prehospital telemedicine system on one specifically equipped ambulance.	not specified	not specified	Ambulance	not specified	264 missions(113 with photographic transmissions)	On-scene time : 23 minGood quality pictures : 100%Failure : 25%
Ter Avest	2019	Prospective feasibilty study	to describe the acceptability and feasibility of the use of live video footage as a dispatch aid for HEMS dispatchers.	Defined as:1. Acceptability for emergency callers to use video transmission 2. Feasibility of the HEMS dispatch system to use video footage	GoodSAM	Patients / Bystander	not specified	21 calls	Acceptability : 100%Failure : 10%Ease to use ; 4.95/5
Pineau	2020	Prospective pilot study	to assess the usefulness and the EMS physician perception of a real-time videoconferencing solution.	Usefullness (Likert scale 0-10)	XpertEye®	healthcare professional / bystander	Not specified	152 video-calls	Usefullness 7/10Able to change decision 43%Failure 26%
Han	2020	Prospective observational study	to ﬁnd out if videotelephony-assisted medical direction (VAMD) can change the intervention of the emergency medical technician compared to using conventional voice calls.	not specified	Android video conference software	Emergency medical technicians	Not specified	312 (131 voice calls / 179 video calls)	Standard vs. Video callsAsk to physician for hospital selection 3% vs 36% ; p < 0,001Dispatching time : 2 (1 - 3) versus 2 (1 - 4) - NS
Linderoth	2021	Retrospective study	to investigate whether live streaming using bystander’s smartphone to the medical dispatcher could improve the quality of bystander CPR in real OHCA cases.	not specified	GoodSAM Instant-on-Scene	Bystander	1. OHCD-suspected calls2. Two bystanders present	90 calls	Change after video-assisted intervention-Improved hand position (60.5%)-Improved compression rate (75%)-Improved compression depth (57.9%)
Linderoth	2021	Retrospective study	to assess feasibility and dispatchers’ perceptions and response after adding live video from bystanders in emergency calls	change in the dispatchers’ emergency response after adding live video	GoodSAM Instant-on-Scene	Bystander	1. Emergency call to 1122. Caller was by the patient's side3. estimated age <18 years4. smartphone with a camera	1020 video calls	Change in response : 51,1% of callsFailure 17,8%Duration of call 423s versus 242s - p < 0,001
Idland	2022	Qualitative study	to explore the dispatchers’ experiences with the use of video streaming of medical emergency calls.	not specified	not specified	none	Dispatcher	25 dispatchers	Improved severity assessmentCaller reassuranceResource allocation optimisationEmotional burdenEthical concerns
Magimel-Pelonnier	2022	prospective, single-centre, randomised-controlled trial	to measure the impact of ambulance staff tele-transmitted photography on prehospital dispatching optimisation for patients calling the EMCC with MTI.	proportion of patients dispatched elsewhere than to the nearest hospital	Photographic transmissionNomadeec®	paramedics	Moderate traumatic injury	165 patients	Standard vs. Photographic transmission-Orientation elsewhere than nearest hospital : 11% versus 33% (OR = 3,80 [1,63-8,90]-Incorrect triaging : 16% versus 7% - NS* (unpublished data)-Under-triage : 16% versus 7% - NS-Over-triage : 25% versus 7% - NS
Marjanovic	2023	Prospective before-after study	to assess how telemedicine software may improve identification of symptoms of severe respiratory failure in patients calling an Emergency Medical Call Center for dyspnea.	proportion of symptoms of severe respiratory failure sought by the emergency physician during the call	Urgentime	Patients / Bystander	1. age ≥ 18 years2. acute dyspnea	150 patients	Standard (before) vs Video-call (after)-Severity symptoms collected 1,9+/-1,1 versus 3,9+/-0,9 - Estimated difference 1,7 [1,4 - 2,0]-Call duration : 4 min vs. 7 min, p < 0,001-No difference on decision at first call-Failure : not reported
Marjanovic	2023	Prospective observationnal study	To compare the proportion of nursing home residents dispatched to an emergency department (ED) after a call to the emergency medical communication center (EMCC) according to the availability or nonavailability of telemedicine.	proportion of residents dispatched to an ED after their ﬁrst call	Nomadeec®	Nurses / assistant nurses	1. age ≥ 18 years2. living in a nursing home	3103 calls (355 in equipped nursing home / 2748 in unequipped nursing home)	Unequipped vs Equipped nursing home-Transfert to an ED : 50% vs 41%, OR=0.72 [0,57 - 0,91]-Day-30 Mortality : 9% vs. 8%, OR 0,86 [0,55 - 1,29]
Ulvin	2023	observational before–after study	to evaluate the impact from VC in the EMCC on HEMS dispatch precision.	proportion of seriously ill or injured HEMS patients	Hjelp 113 Video	Patients / Bystander	Missions HEMS Trondheim	unclear	Standard (before) vs. Video (after)-Patients with Severe illness (NACA 4-7) : 70% versus 75% - NS-Inappropriate HEMS dispatching : 40,3% vs 28.4% - p = 0,007-Failure : 6,1%
Gren	2023	Prospective observational study	To investigate if the new triage tool video triage resulted in a higher proportion of patients staying at home the next 8 h after the call	Proportion of patients staying at home	GoodSAM	Parents	1. age between 3 month and 5 years 2. with fever	754 calls (371 in video triage group / 383 in telephone triage group)	Video vs telephone : - Patients assessed at hospital 47% vs 45%, p = 0.58 - Failure 5%
Gren	2023	Prospective observational study	To study the safety and feasibility of introducing video triage of young children with respiratory symptoms	number of patients staying at home for eight hours after the call	GoodSAM	Parents	1. age between 6 month and 5 years 2. with respiratory symptoms	617 calls (336 in video triage group / 281 in telephone triage groupà	Video vs telephone : - Patients assessed at hospital 39% vs 46%, p = 0.07 - Failure 2.4%
Taylor	2024	Feasibility randomised controlled trial	to assess the feasibility of implementing and evaluating GoodSAM Instant-On-Scene	decision regarding the feasibility	GoodSAM	Patients / Bystander	1. Trauma patients 2. Laystander caller	244 incidents -standard 132 -video-call 108	Acceptation : 86% Failure : 15%
Idland	2024	prospective observational study	to investigate if video streaming is associated with recognition of a need for first aid during calls regarding injured patients and improve quality of bystander first aid.	whether the dispatcher recognized a need for bystander first aid or not during the medical emergency call.	Not reported	Ambulance	1. Patient who had an injury2. One or more bystander presents3. Aid measures requirements	113 patients 12 video-calls	Video-calls vs. Standard-calls-First-aid needing recognizing : 83% vs 49% - OR 5.30; 95% CI [1.11–25.44]-High-quality care : 83% vs. 73% - p = 0,10
Nanou	2024	prospective, single-centre, randomised-controlled trial	to assess the impact of telemedicine through caller smartphone camera on the dispatching of patients with mild traumatic injury.	proportion of patients dispatched to settings other than the nearest hospital	Nomadeec	Patients / Bystander	1. age ≥ 18 years2. mild traumatic injury	153 patients76 standard calls77 video calls	Standard vs. Video-call-Orientation elsewhere nearest hospital : 22% versus 46%, Difference = 24 [10 - 39]-Incorrect triaging : 3% versus 1% - NS (unpublished data)-Under-triage : 0% versus 3% - NS-Over-triage : 2% versus 3% - NS-Failure : not reported
Accorsi	2025	randomized, noninferior, open-label study.	to examine the practicality and preliminary effects of acquiring medical information via teleconsultation during ambulance dispatches on the duration of a prehospital team’s stay at the care site	length of stay in minutes from the ambulance parking on the scene to the ambulance departure from the patient for the emergency facility.	WhatsApp	Patients / Bystander	1. age ≥ 18 years2. exclusion of trauma and cardiac arrest3, exclusion of patients without WhatsApp application	20 patients10 video calls10 standard calls	Video vs. Standard calls-On-site length of stay 20+/-6 vs 37+/-20 - p = 0,019
Idland	2025	Qualitative study	to explore the caller’s experiences with video streaming.	not specified	none	Callers	1. called the medical emergency number 113 in the Oslo region 2. video streaming had been used during the call.	245 survey respondents	Increased sense of safetySurprise regarding the technologyEasier communicationEmotional discomfortIncreased stress
Faurholdt Gude	2025	cluster randomized clinical study	To assess whether video streaming during emergency calls reduces highest-urgency ambulance dispatches	percentage of ambulances dispatched at the highest urgency level	Dynamic Infrastructure for Applications and Services	Patients / Bystander	1. Calls to 112	8124 in video-call cluster [3706 video-streaming established] 10621 in standard cluster	Video-call cluster vs. Standard cluster-Highest level of urgency rate : 33 (30 - 37) vs 38 (35,5 - 45) - Difference -5% [-10 to 0]-Mortality : 2,7% vs 2,7% - NS-Call duration : 4,1 min vs 3,8mn - Difference 0,5 min-Failure : 29%

*EMS* Emergency Medical Services, *HEMS* Helicopter Emergency Medical Services, *NIHSS* National Institutes of Health Stroke Scale, *NS* Not Significant, *VAMD* Videotelephony-Assisted Medical Direction, *VC* Video Call, *CI* Confidence Interval, *ED* Emergency Department, *EMCC* Emergency Medical Communication Center, *NACA* National Advisory Committee for Aeronautics, *OR* Odds Ratio, *CPR* Cardiopulmonary Resuscitation, *MTI* Moderate Traumatic Injury, *OHCA* Out-of-Hospital Cardiac Arrest

Among them, 2 (11%) had a qualitative design and 3 (15%) were feasibility trials. The remaining clinical studies included 6 (30%) prospective observational studies, 5 (25%) randomised controlled trials, 2 (10%) retrospective studies and 2 (10%) before–after studies.

Two qualitative studies included dispatchers or callers to explore, through interview or standard questionnaire, the usability and acceptability of telemedicine from the users’ perspective [13, 14]. In addition, 18 clinical studies involved heterogeneous patient populations to assess the impact of image transmission or video-live-streaming: 11 included non-specific patient cohorts [12, 15–24], 2 focused on minor trauma cases [10, 11], 2 on children [25, 26], 1 on older adults [27], 1 on cardiac arrest [28], and 1 on patients with acute dyspnoea [29].

The impact on triage quality was reported in 3 (15%) studies [10, 11, 21], on dispatch decision-making in 8 (40%), [10–12, 19, 25–27, 29] on situation assessment in 2 (10%) [24, 29], on primary care by bystanders in 2 (10%) [20, 28], on call duration in 6 (30%) studies [12, 19, 20, 23, 25, 29], and on technical failure rate in 6 (30%) studies. Ethical considerations related to image transmission and video live-streaming were not specifically addressed in any of the included studies, but one assessed risk of psychological harm [30].

### Feasibility, usability and acceptance

EMCCs are tasked with rapidly assessing callers’ situation by phone, considering their location, the level of urgency, and the available resources for assistance or transport. In the context of rising daily call volumes and increasing time pressure, evaluating the feasibility and acceptability of visual support by either the caller or the dispatcher —whether through photo transmission or video-assisted triage—is a key priority.

Literature data suggest the feasibility of video live-streaming based triage and image transmission in EMCCs, with several studies demonstrating its successful integration across various settings. Video live-streaming appears usable by medical dispatchers, paramedics [15, 31] or patients [14, 17, 32], as well as image transmission [15, 16]. Healthcare professionals generally perceive this system as effective, as it provides valuable additional information and influences medical dispatch decisions [13, 33]. Video live-streaming is reported to be easy to use and enhances visual assessment of the situation, particularly when the information provided by the caller is unclear [32, 34]. Image transmission provides high quality pictures, relevant to take a dispatching decision [15]. Video live-streaming based triage has been shown to support critical decisions, such as allowing patients to remain at home with appropriate advice [35], reassessing the need for helicopter emergency medical services [17], or guiding technical procedures such as cardiopulmonary resuscitation (CPR) [20, 36]. However, video live-streaming has not been associated with any change in patient disposition when the call concerned a child with fever or dyspnoea [25, 26]. In addition, some professionals indicate that video is not useful in every situation and may sometimes make the regulation process uncomfortable. Dispatchers reported greater emotional discomfort when children were involved, although this varied depending on their clinical experience (Table 2) [13].

**Table 2:** Summary of benefits and disadvantages when using photo transmission or video-live streaming in an Emergency Medical Communication Centre

Domain	Benefits	Disadvantages
Dispatch decisions	• Reduction of inappropriate dispatches • Reduction of unnecessary ED transfers • Possibility to safely keep patients at home with adequate advice	• Prolonged call duration • Variable success rate
Bystander first aid	• Improved CPR quality (hand position, frequency, depth) • Increased recognition of first aid needs	• May induce emotional stress for callers
Feasibility and acceptability	• High acceptance among callers and dispatchers • Strengthens emotional connection, reduces feeling of isolation	• Possible discomfort for some dispatchers • Requires informed consent, sometimes difficult in emergencies
Organisation and equity	• Potential to optimise resource allocation • Supports dispatcher training in visual assessment	• Risk of widening digital divide (poor coverage, old devices) • Ethical/legal concerns (confidentiality, risk of “tele-negligence”)

*ED* Emergency Department, *CPR* Cardiopulmonary resuscitation

While image transmission or video-live-streaming based triage hold promise in urgent care contexts, patient acceptability remains a key factor. Only four studies have assessed it, reporting high acceptance rates from 86 to 100% [16, 17, 25, 26, 34, 35]. Furthermore, videoconferencing during emergency calls creates a perceived presence of the dispatcher, reducing feelings of isolation among callers [13, 14]. Some callers reported reassurance from no longer feeling alone in decision-making. Even without visual feedback, the knowledge that the dispatcher can see the scene enhances emotional connection and a sense of safety [14].

### Operational aspects

The use of image transmission and video live-streaming in the context of emergency medical dispatch is generally based on dedicated applications. When relying on the caller’s smartphone, these solutions must comply with strict specifications: no need for prior download, no account creation, maintenance of the communication with the EMCC, no storage of personal data, no access to the phone’s content, and transmission via a secure and compliant platform.

The main solutions reported in published studies — XpertEye (Advanced Mobile Application, London, UK), GoodSAM (GoodSAM Instant.Help, London, UK), Urgentime (Urgentime, Lyon, France), Nomadeec (Ennovacom, Bordeaux, France), Hjelp 113 video (Videoløsningen Hjelp 113 Video, Norsk Luftalbulance, Drøbak, Norway) — meet these requirements. They operate through the sending of an SMS hyperlink, which the caller activates after providing explicit consent via a dialog box, or by granting camera access authorisation, which is considered equivalent to consent [10, 11, 17, 18, 20, 28, 29]. This enables live access to the smartphone camera and real-time video transmission to the dispatch physician, without interrupting the ongoing call. The video stream is transmitted from the caller to the EMCC, but not in the reverse direction, meaning that callers generally do not see their interlocutor. Other studies have proposed alternative approaches, involving physicians, paramedics or nurses equipped with dedicated devices [10, 27]. Although rarely evaluated, this strategy could represent a relevant option in specific situations — particularly when the patient is unable to use a phone, when healthcare providers are present, or when emergency services have been dispatched as a precaution. Such systems may also offer additional advantages, including access to a summarised patient record or the possibility of performing an ECG remotely.

Some studies mention the use of non-dedicated applications such as WhatsApp or Androïd specific software [19, 23]. Although technically functional, these options raise significant ethical and legal concerns: they require the application to be pre-installed and do not offer any guarantee regarding data security, privacy, or compliance with personal data protection regulations.

### Clinical applications and impact on triage

The reasons for which video is used during emergency calls vary considerably across studies. It is primarily employed in cases of included unspecified calls, altered consciousness, seizures, road traffic accidents [12, 20], minor trauma [10–12], dyspnoea [29], nursing home residents [27], or when children are involved [12, 25, 26]. Clinical situations that are either non-severe or, conversely, extremely severe are less frequently associated with the use of visual support, except when it is used to guide first aid interventions.

The actual impact of visual support use remains unclear. On situation awareness, video live-streaming may enhance the assessment of the on-scene situation and facilitate the guidance of bystander interventions [24, 28]. However, these findings are mostly based on self-reported data from medical dispatchers. Two studies have assessed the quality of symptom collection by dispatchers. In a quasi-experimental before-and-after study, use of video live-streaming was associated with more accurate evaluation of respiratory distress symptoms, and more comprehensive and structured symptom reporting [29]. These findings suggest that adding visual support enables a more accurate assessment of the situation than discussion alone, potentially comparable to an in-person clinical examination.

**Fig. 2:**
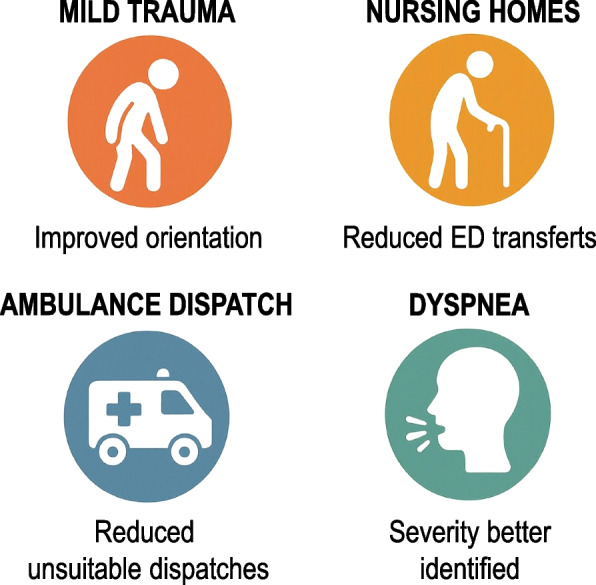
Clinical situations assessed in the litterature

However, the primary purpose of using visual support should be to reduce the risks of under-triage and over-triage through improved situation assessment. Under-triage and over-triage rates reflect the quality of triage performed by the medical dispatcher. These are critical indicators, given their impact on patient outcomes and the organisation of the healthcare system. Nevertheless, quality of triage has been rarely evaluated, with most studies focusing on patient classification and disposition. In a single centre study, use of video reduced inappropriate HEMS dispatching, without addressing other triage decisions (Fig. 2) [21].

Two other single-centre, randomised, controlled trials focusing on minor trauma also reported the use of image transmission and video-live streaming to be associated with improvement in patient disposition: provision of medical advice, and transfer to higher-level hospital for the most severe cases [10, 11]. However, neither image transmission nor video streaming has been associated with a reduction in under-triage or over-triage, which remained infrequent across all groups. In addition, two studies focused on dispatching decision, without triage quality assessment, after using video live-streaming and/or image transmission. In a single-centre stepped-wedge cluster randomised trial, video live-streaming–based triage was associated with changes in patient severity classification, regardless of the reason for the call, and reduction in high-level ambulance dispatches (–5%, 95% CI –10% to 0%) [12]. Despite a large sample size (n = 18,745), only 64% of patients in the video group had video connection attempt, with success rate of 71%. As a result, the true impact of video live-streaming was likely underestimated. Finally, an observational study conducted in 74 nursing homes reported an 18% reduction in ED transfers following EMCC calls when video or photos could be transmitted, without increasing the number of callbacks or mortality [27]. However, the clinical situations in which these tools were used were not clearly described, and the actual impact of visual support remains unclear (Fig. 2).

### Limitations and technical failures

While these tools offer promising advantages, it is crucial to highlight their operational constraints within EMCCs. First, whether involving the transmission of photographs or the use of video live-streaming, medical triage time is frequently extended. Although the literature suggests this added time is modest [12, 19, 25, 29], its cumulative impact across the high daily call volume in an EMCC may significantly affect overall performance and responsiveness (Table 2) [12].

A second limitation is the risk of technical failure, more frequent with video live-streaming than still image transmission. Reported failure rates are substantial and range from 2.4% to 30%, depending on the study [12, 15, 18–21, 25, 26]. Common causes for failure include insufficient mobile or wireless coverage, device incompatibility, or callers struggling to follow technical instructions. These challenges highlight the persistence of a digital divide and raise concerns about inequitable access to emergency care, influenced not only by the type of phone patients own, but also by their geographic location [37].

Finally, obtaining informed consent from the caller remains a critical challenge. This step may lead to delays, confusion, or outright refusal, particularly in high-stress situations or when the caller is not the patient. To streamline the process, consent is generally implied, as callers authorise the activation of their camera and the transmission of video. The ethical and legal use of remote visual technologies requires well-defined, flexible protocols that can accommodate the diverse and urgent realities of emergency care [38].

### Ethical and legal considerations

Ethical considerations surrounding the use of telemedicine, including video-assisted dispatching, remain poorly studied and highly heterogeneous. Existing literature suggests that specific informed consent should be obtained; however, this is not consistently implemented in practice. In real-life situations, though, patients or callers generally provide tacit consent simply by activating the camera function. Moreover, the psychological impact of using video on callers must be considered, as it can be distressing to film someone in a state of distress [30].

## Discussion and perspectives

Data from studies highlighted in this review suggest that use of image transmission and video live-streaming is both feasible and well accepted by dispatchers and callers. Such visual tools appear to enhance dispatchers’ situational awareness and may influence final decision-making. However, few studies have specifically evaluated the impact of visual support on the quality of triage, and few in specific clinical situations. Thus, studies specifically assessing the impact of these tools on triage, evaluated in a pragmatic and clinically relevant manner and in clearly defined situations, are needed. An ongoing study is expected to provide such results in the coming years (e.g., see NCT06847997).

Despite promising results, several aspects remain unclear and require further development. On an operational level, the failure rate is still too high [12, 18]. Future technological advancements should aim to reduce this risk of failure, speed up connection times, and improve the quality of transmitted photos and videos. At the same time, the fight against geographical inequalities remains a crucial issue. The existing digital divide is likely to widen further, thus increasing disparities in access to pre-hospital care [39].

Regarding the evolution of practices, the use of video support will have a significant impact on the training and skills of medical dispatchers. They will need to be trained not only in assessing situations via video but also in managing the differences between video evaluation, telephone assessment, and real-life evaluation. Visual support could be crucial in facilitating decision-making beyond the threshold of uncertainty [40]. However, it will be essential to clearly define the situation in which video use is relevant. Further research, targeting specific call reasons, is needed to avoid inappropriate use, which could both affect patient care and disrupt the organization of the EMCC by prolonging call durations.

Nevertheless, a further major challenge lies in the practical implementation of these technologies. Although image transmission and live video are now technically available in many EMCCs, their routine use remains uncommon. This implementation gap underscores the contrast between demonstrated feasibility and actual integration into everyday dispatch workflows. Visual tools are still often regarded as optional rather than embedded in standard operating procedures. Establishing clear best practices and dedicated training frameworks for medical dispatchers will therefore be essential to ensure their effective, consistent, and safe use.

Additionally, the organisational and economic impact should be evaluated to ensure sustainable and standardised implementation of such systems. These factors may represent significant barriers to implementation and to the broader deployment of the system across entire counties.

Then, misuse of visual support in EMCCs must also be considered. Authors have introduced the concept of tele-negligence, referring to medical errors attributable to the use of telemedicine, for which liability in the event of litigation remains unclear [38]. Although empirical data on this phenomenon are limited, the associated risks should not be overlooked. However, these aspects have not yet been explored in the literature. Legal and regulatory frameworks are so essential. In France, the use of video in EMCC is considered part of telemedicine and is regulated by the Haute Autorité de Santé (French High Authority of Health) [8, 9, 41]. Regardless of the setting, the use of video—and telemedicine more broadly—requires oversight by health authorities to prevent misuse.

Finally, from an ethical aspect, the American College of Emergency Physicians (ACEP) has issued several recommendations, notably emphasising that systems must ensure level of security sufficient to guarantee confidentiality equivalent to that of a standard clinical examination. Similarly, access to video live streaming based triage should not be influenced by race, religion, sexual orientation, geographic location, or socio-economic status. These systems must adhere to the principles of informed consent, and in all cases, the care provided should be guided by the patient’s health needs [39].

## Limitations

This review suffers from limits. First, it is based on a narrative review, which, although suitable for integrating heterogeneous evidence and contextual insights, does not follow a fully systematic or reproducible search strategy. Moreover, the selected studies cover a wide range of clinical contexts and use cases, from remote clinical assessment to the guidance of technical procedures. Although these situations share the same underlying principle of remote visual evaluation, they correspond to distinct operational and clinical settings with different objectives. This heterogeneity therefore limits both the comparability of results and their extrapolation to specific scenarios that have not yet been explored in the literature.

## Conclusion

Image transmission and video live-streaming for emergency triage appear feasible and promising in EMCC. It is generally accepted by medical dispatchers, paramedics, and patients, who consider it useful. This approach may improve decisions made by medical dispatchers. However, its impact on the quality of triage still needs to be more formally assessed.

## Data Availability

No datasets were generated or analysed during the current study.
